# Supplementary material for: The Role of Type 2 Diabetes in Patient Symptom Attribution, Help-Seeking, and Attitudes to Investigations for Colorectal Cancer Symptoms: An Online Vignette Study
Source: Cancers (Basel). 2023 Mar 8;15(6):1668. doi: 10.3390/cancers15061668 (PMC10045970; doi:10.3390/cancers15061668)
Supplement: Supplementary file 1 [file cancers-15-01668-s001.zip › cancers-2219345-supplementary.pdf]

SA: Vignette Questionnaire

**Vignette**

**Please read the following scenario and imagine you were in that situation.**
***Scenario***

*You notice you have [symptom].*

***What do you think the cause of [symptom] could be? Please write in the box below as many things as come to your mind. If you cannot think of any please write 'don't know' in the box.***

***What actions would you take if you were to notice [ symptom] as described in the scenario above? Next to each option, please tick the box that best reflects how likely you are to take each action.***

| | **Definitely would** | **Probably would** | **Probably wouldn't** | **Definitely wouldn't** | **Not applicable** |
|---|---|---|---|---|---|
| Talk to members of your family about the symptoms? | ☐ | ☐ | ☐ | ☐ | ☐ |
| Go to the pharmacy (chemist) for advice | ☐ | ☐ | ☐ | ☐ | ☐ |
| Contact the GP about the change in [symptom] | ☐ | ☐ | ☐ | ☐ | ☐ |
| Mention the symptoms if you saw the GP for another reason | ☐ | ☐ | ☐ | ☐ | ☐ |
| Go to A&E | ☐ | ☐ | ☐ | ☐ | ☐ |
| Look up information about the symptoms online | ☐ | ☐ | ☐ | ☐ | ☐ |
| Wait and see what happens (e.g., if the symptoms get worse) | ☐ | ☐ | ☐ | ☐ | ☐ |
| Dismiss the symptoms as something not to worry about | ☐ | ☐ | ☐ | ☐ | ☐ |
| Contact a nurse about the symptoms | ☐ | ☐ | ☐ | ☐ | ☐ |
| Mention the symptoms if you saw a nurse for another reason | ☐ | ☐ | ☐ | ☐ | ☐ |
| Contact a diabetes specialist | ☐ | ☐ | ☐ | ☐ | ☐ |
| Contact an endocrinologist | ☐ | ☐ | ☐ | ☐ | ☐ |
| Other | ☐ | ☐ | ☐ | ☐ | ☐ |
| If **'other'** please specify: | | | | | |

***Scenario b:***

| *Imagine you have decided to see your GP about[ symptom], an appointment has been made.* |
|---|

***Question only for vignette 3: In which order would you choose to mention these symptoms to the GP?***

| ***Order of preference*** | |
|---|---|
| | Numbness, tingling and pain in feet |
| | Rectal Bleeding |
| | Change in bowel habits |

***How would you tell the doctor about these symptoms? Please tell us in your own words what you would tell the doctor, expanding to also describe how you might feel about these symptoms.***

| What would you say:<br><br>How would you feel: |
|---|

***Would you mention any underlying health conditions you may have?***

| ***Yes*** ☐ | ***No*** ☐ | ***If the GP asked*** ☐ |
|---|---|---|

***Scenario c***

| *Still imagine that you have been experiencing [symptom of bowel changes] you have now spoken to a GP. They have suggested you perform a stool test. (A stool sample involves collecting a small amount of faeces in a container at home and returning it to your GP/hospital).* |
|---|

***Would you be willing to partake in this?***

| ***Yes*** ☐ | ***No*** ☐ |
|---|---|
| ***If no, please give a reason:*** | |

***Do you think your actions would have been different pre-Coronavirus?***

| ***Yes*** ☐ | ***No*** ☐ |
|---|---|
| ***If yes, please give a reason:*** | |

***Scenario d:***

| |
|---|
| *Still imagining that you have been experiencing [symptom of rectal bleeding], you have now spoken to a GP and they have suggested to refer you for a colonoscopy/sigmoidoscopy. (A colonoscopy/sigmoidoscopy use a thin flexible tube with a camera on the end to look at the colon).* |

***Would you be willing to partake in this?***

| | |
|---|---|
| ***Yes*** ☐ | ***No*** ☐ |
| ***If no, please give a reason:*** | |

***Do you think your actions would have been different pre-Coronavirus?***

| | |
|---|---|
| ***Yes*** ☐ | ***No*** ☐ |
| ***If yes, please give a reason:*** | |

***How comfortable would you be in attending the hospital, please write down any feeling or concern you may have about attending?***

| |
|---|
| |

***Do you think your level of comfort in visiting a hospital has changed due to COVID-19?***

| | |
|---|---|
| ***Yes*** ☐ | ***No*** ☐ |
| ***Please expand:*** | |

SB : Symptom attribution table

Symptom attribution by type 2 diabetes based on answers to the three vignettes

| | Total % | With diabetes | Without diabetes | P value (χ2 test) |
|---|---|---|---|---|
| **Rectal Bleeding** | | | | |
| Cancer | 377 (29.3%) | 86 (22.8%) | 291 (30.1%) | .268 |
| Haemorrhoids/Piles | 382 (29.7%) | 95 (29.7%) | 287 (29.7%) | .998 |
| Damage to anus inc. Anal tear/fissure | 102 (7.9%) | 14 (4.4%) | 88 (9.1%) | .007 |
| Constipation/straining | 87 (6.8%) | 20 (6.3%) | 67 (6.9%) | .675 |
| Bowel disease inc. IBS | 60(4.7%) | 16 (5.0%) | 44(4.6%) | .741 |
| Don't know | 55 (4.3%) | 16 (5.0%) | 39(4.0%) | .461 |
| | | | | |
| **Changes in Bowel Habit** | | | | |
| Cancer | 185 (14.4%) | 31 (9.7%) | 154 (15.9%) | .006 |
| Dietary Changes | 430 (33.4%) | 99 (30.9%) | 331 (34.2%) | .274 |
| Bowel disease inc. IBS | 147 (11.4%) | 26 (8.1%) | 121 (12.5%) | .032 |
| Stomach Infection/food poisoning | 128 (9.9%) | 31 (9.7%) | 97 (10.0%) | .859 |
| Don't know | 57 (4.4%) | 19 (33.3%) | 38(3.9%) | 0.130 |
| Caused by (change in) medication | 47 (3.7%) | 23 (7.2%) | 24 (2.5%) | <0.001 |
| | | | | |
| **Rectal Bleeding, Changes in Bowel Habit and Numbness and Pain in the Feet** | | | | |
| Cancer | 471 (36.6%) | 110 (34.4%) | 361 (37.3%) | .341 |
| Haemorrhoids/ Piles | 179 (13.9%) | 46 (14.4%) | 133 (13.8%) | .781 |
| Bowel disease inc. IBS | 148 (11.5%) | 41 (12.8%) | 107 (11.1%) | .396 |
| Dietary Changes | 86 (6.7%) | 20 (56.3%) | 67 (6.9%) | .675 |
| Don't know | 51 (4.0%) | 14 (4.4%) | 37 (3.8%) | .663 |
| Diabetes | 46 (3.6%) | 11 (3.4%) | 35 (3.6%) | .879 |

(Columns may equal >100% due to multiple answers per participant being given)

SC : Symptom attribution Logistic regression figures for vignette 1 and 3.

*Logistic regression odds ratios and 95% confidence intervals for symptom attributions when experiencing rectal bleeding (vignette 1) reported by participants with diabetes compared to those without (adjusted for other comorbidities, age, sex, and ethnicity). Each symptoms attribution was included as the outcome (binary yes/no outcome) in a separate multivariable model.*

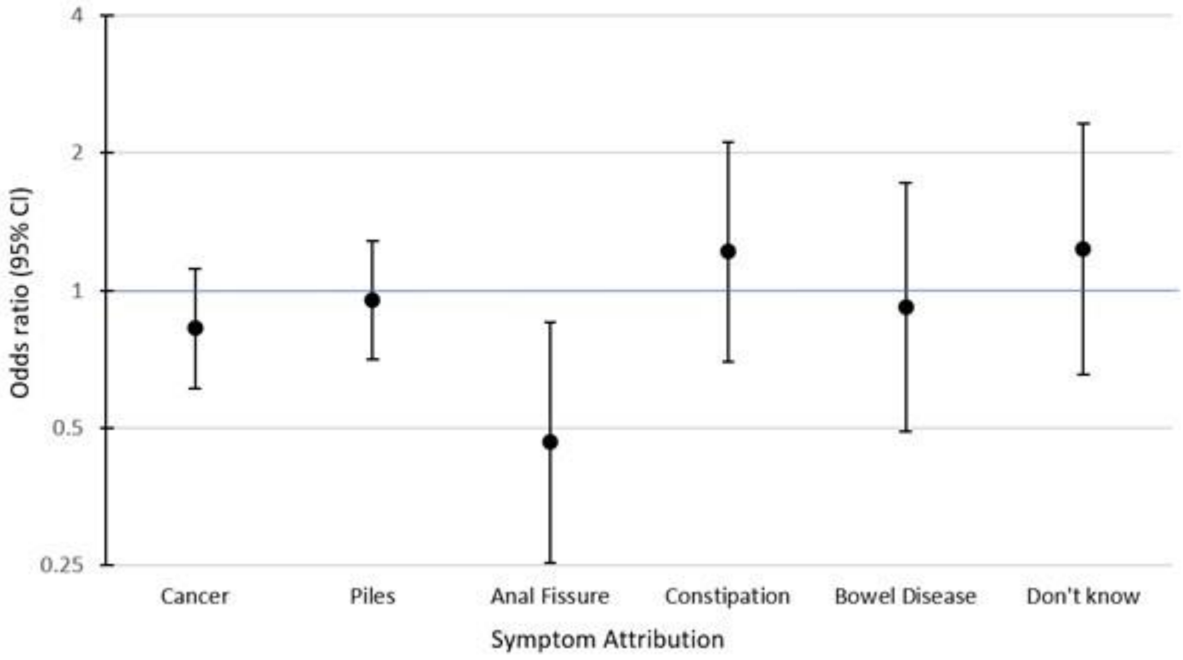

*Logistic regression odds ratios and 95% confidence intervals for symptom attributions when experiencing rectal bleeding, change in bowel habits and numbness. tingling in feet (vignette 3) reported by participants with diabetes compared to those without (adjusted for other comorbidities, age, sex, and ethnicity). Each symptoms attribution was included as the outcome (binary yes/no outcome) in a separate multivariable model.*

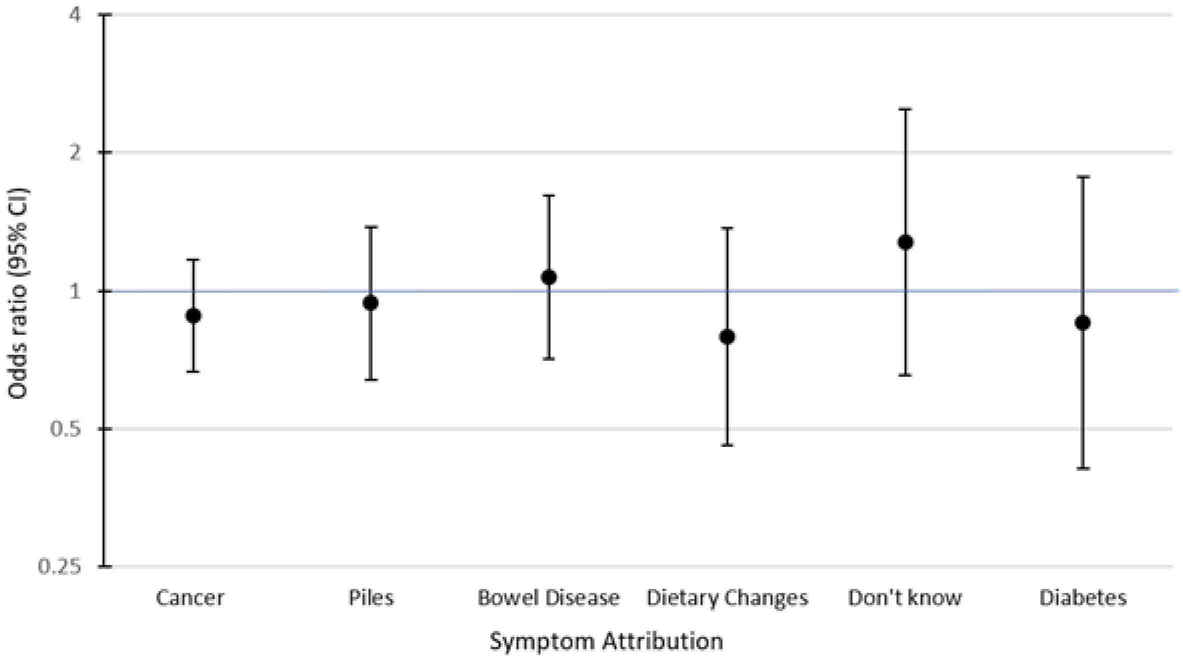

SD: Willingness to undergo diagnostic testing for new symptoms (rectal bleeding or change in bowel habit) by diabetes status, by previous testing history, age, sex, ethnic group, and comorbidities. (N = 1282)

| | **Stool sample** | **Colonoscopy/Sigmoidoscopy** |
|---|---|---|
| *Unadjusted models* | **OR (95% CI)** | **OR (95% CI)** |
| **Diabetes** | | |
| Yes | 1.21 (0.47-3.15) | 1.01(0.54-1.87) |
| No | 1.0 | 1.0 |
| | | |
| *Adjusted models* | | |
| **Diabetes** | | |
| Yes | 0.94(0.33-2.67) | 0.99(0.51-1.92) |
| No | 1.0 | 1.0 |
| | | |
| | | |
| **Previous colonoscopy/sigmoidoscopy** | N/A | |
| Yes (for screening or symptoms) | | **0.28(0.12-0.63)**** |
| No | | 1.0 |
| | | |
| **Previous Stool test** | | N/A |
| Yes (for screening or symptoms) | **0.16 (0.04-0.35)***** | |
| No | 1.0 | |
| | | |
| **Sex** | | |
| Male | 1.39 (0.56-3.47) | **0.37(0.19-0.73)**** |
| Female | 1.0 | 1.0 |

*=<0.05 **<=0.01 ***<=0.001

SE : Main themes of participants who responded no to partaking in diagnostic testing table

Main themes of participants who responded no to partaking in diagnostic testing.

| | Total participants N=21 | With diabetes N = 6 | Without diabetes N = 15 |
|---|---|---|---|
| ***Stool sample*** | | | |
| Embarrassing/Scared | 4 | 1 | 3 |
| Unnecessary | 4 | 1 | 3 |
| Physically difficult | 3 | 0 | 3 |
| Unconcerned that there is an issue | 1 | 0 | 1 |
| Unpleasant | 4 | 2 | 2 |
| ***Colonoscopy*** | | | |
| | **Total participants N=56** | **With diabetes N = 14** | **Without diabetes N = 42** |
| Embarrassing/Anxiety/Nervous | 15 | 2 | 13 |
| Too extreme/ invasive | 8 | 1 | 7 |
| Uncomfortable/painful | 12 | 3 | 9 |
| COVID/Hospital concerns | 6 | 1 | 5 |
| Would try alternatives first | 4 | 0 | 4 |
| Would insist on being sedated/ under GA | 4 | 0 | 4 |
| Negative previous hospital/procedure experience | 4 | 2 | 2 |

SF: Symptoms mentioned by participants as the first priority to talk to the GP about by diabetes status. (N = 623)

| | Total participants mentioning symptom as 1st priority N = 623 | With diabetes N = 157 | Without diabetes N = 467 | P value (χ2 test) |
|---|---|---|---|---|
| **Rectal Bleeding** | 461 (74.2%) | 103 (65.6%) | 359 (77.0%) | 0.004 |
| **Change in bowel habits** | 62 (10.0%) | 16 (10.3%) | 46 (9.9%) | 0.871 |
| **Numbness/pain in feet** | 100 (16.1%) | 38 (24.2%) | 62 (13.3%) | 0.001 |
